# Role of G Protein-Coupled Receptors in Hepatic Stellate Cells and Approaches to Anti-Fibrotic Treatment of Non-Alcoholic Fatty Liver Disease

**DOI:** 10.3389/fendo.2021.773432

**Published:** 2021-12-06

**Authors:** Takefumi Kimura, Simran Singh, Naoki Tanaka, Takeji Umemura

**Affiliations:** ^1^ Molecular Signaling Section, Laboratory of Bioorganic Chemistry, National Institute of Diabetes and Digestive and Kidney Diseases, National Institutes of Health, Bethesda, MD, United States; ^2^ Department of Internal Medicine, Division of Gastroenterology, Shinshu University School of Medicine, Matsumoto, Japan; ^3^ Department of Biological Sciences and Bioengineering, Indian Institute of Technology, Kanpur, India; ^4^ International Relations Office, Shinshu University School of Medicine, Matsumoto, Japan

**Keywords:** hepatic stellate cells (HSC), GPCR (G protein coupled receptor), NAFLD (non-alcoholic fatty liver disease), non-alcoholic steatohepatitis (NASH), liver, fibrosis, metabolism

## Abstract

The prevalence of non-alcoholic fatty liver disease (NAFLD) is globally increasing. Gaining control over disease-related events in non-alcoholic steatohepatitis (NASH), an advanced form of NAFLD, is currently an unmet medical need. Hepatic fibrosis is a critical prognostic factor in NAFLD/NASH. Therefore, a better understanding of the pathophysiology of hepatic fibrosis and the development of related therapies are of great importance. G protein-coupled receptors (GPCRs) are cell surface receptors that mediate the function of a great variety of extracellular ligands. GPCRs represent major drug targets, as indicated by the fact that about 40% of all drugs currently used in clinical practice mediate their therapeutic effects by acting on GPCRs. Like many other organs, various GPCRs play a role in regulating liver function. It is predicted that more than 50 GPCRs are expressed in the liver. However, our knowledge of how GPCRs regulate liver metabolism and fibrosis in the different cell types of the liver is very limited. In particular, a better understanding of the role of GPCRs in hepatic stellate cells (HSCs), the primary cells that regulate liver fibrosis, may lead to the development of drugs that can improve hepatic fibrosis in NAFLD/NASH. In this review, we describe the functions of multiple GPCRs expressed in HSCs, their roles in liver fibrogenesis, and finally speculate on the development of novel treatments for NAFLD/NASH.

## Introduction

**Figure 1:**
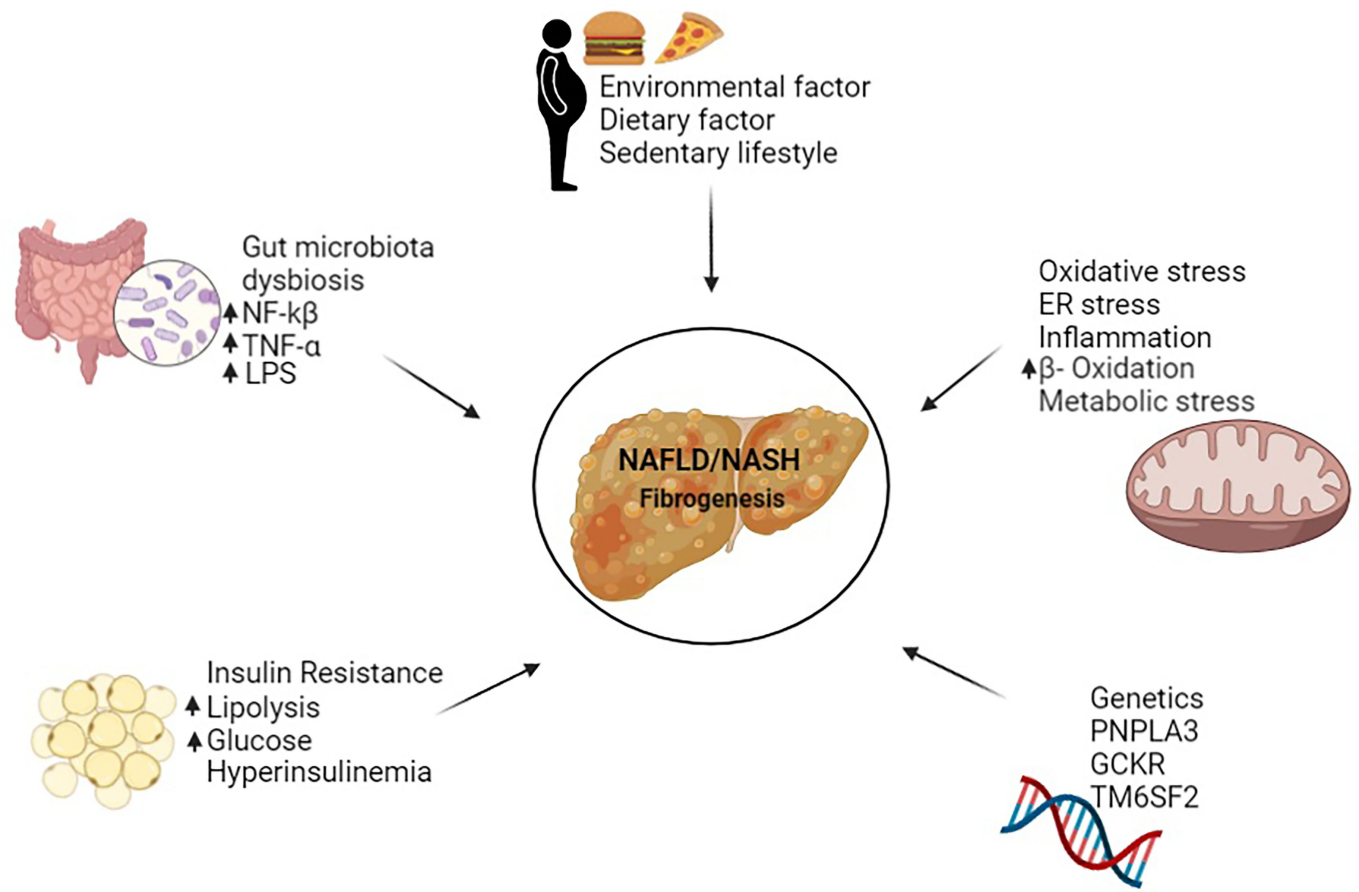
Factors causing NAFLD/NASH in human.

Non-alcoholic fatty liver disease (NAFLD) is a predominant liver disease with a rapid increase in prevalence worldwide, accounting for the hepatic phenotype of the metabolic syndrome (1). NAFLD is a broad disease ranging from simple fatty liver to non-alcoholic steatohepatitis (NASH), advanced fibrosis, cirrhosis, and hepatocellular carcinoma (2). A complex combination of genetic and environmental factors shapes the pathogenesis and stages of NAFLD (1). These factors include patatin-like phospholipase domain-containing protein 3 (*PNPLA3)*, dietary fats, insulin resistance, intestinal bacteria, oxidative stress, endoplasmic reticulum stress, lipotoxicity and immune response (**Figure 1**) (1, 3–5). Among the many factors involved in NAFLD, hepatic fibrogenesis has recently been identified as a prognostic factor in patients with NAFLD (6, 7).

The liver is composed of hepatocytes, biliary epithelial cells, hepatic stellate cells (HSCs), smooth muscle cells, vascular endothelial cells, various immune cells, and sinusoidal endothelial cells (8). Each of these cell types has unique functions and collectively regulates liver function at multiple levels (8). Among these cells, liver fibrosis mainly occurs through the activation of HSCs in various liver diseases, including NAFLD/NASH (1). HSCs reside in the space of Disse between the basolateral surface of hepatocytes and the anti-lateral surface of the fenestrated sinusoidal endothelial cell layer (9). In the space of Disse, biomolecules are exchanged between the portal blood flow from the gastrointestinal tract and the hepatocytes (9). HSCs respond to signals such as cytokines and growth factors from hepatocytes, macrophages, and sinusoidal endothelial cells (9). HSCs are activated by liver injury and become proliferative fibrogenic myofibroblasts, which play the most important role in liver fibrosis (1). Therefore, a better understanding of the function and the regulatory mechanisms of HSCs may prove useful for the treatment of NAFLD/NASH.

**Figure 2:**
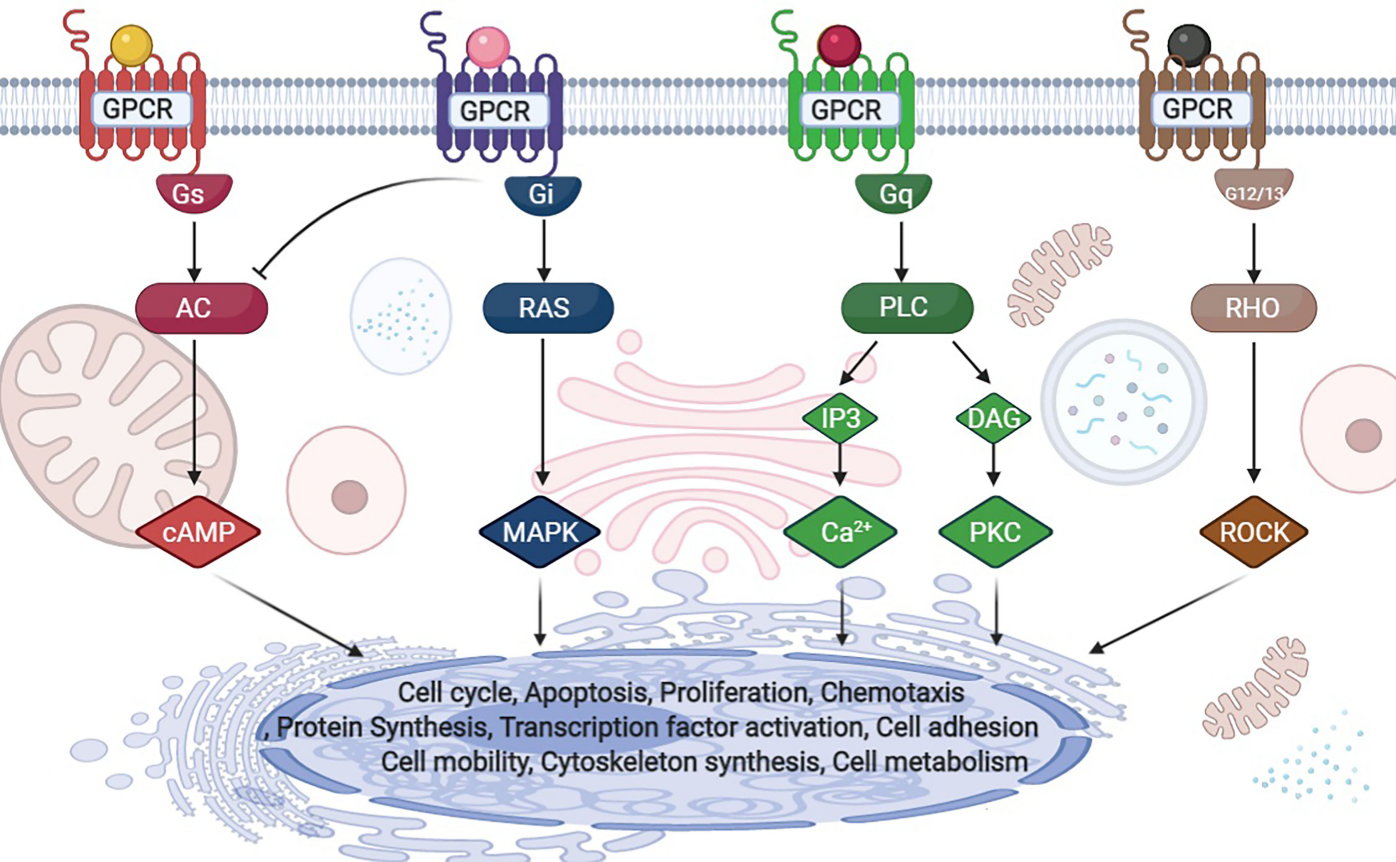
G-protein classification and downstream signals.

G protein-coupled receptors (GPCRs) are cell surface receptors that mediate the function of a wide range of extracellular ligands including, neurotransmitters, secondary metabolites and hormones (10). The human genome contains approximately 800 GPCR genes, accounting for 3-4% of all human genes (11). Approximately 40% of the drugs used in clinical practice exhibit therapeutic effects by acting on GPCRs, thus highlighting the importance of understanding how GPCRs work at the cellular and molecular level (12). Ligand-bound GPCRs recognize and activate heterotrimeric G proteins comprising Gα, Gβ, and Gγ. G proteins are classified into four families according to their α subunits: Gs, Gi, Gq, and G12/13 (**Figure 2**) (10). Gs and Gi regulate adenylyl cyclase activity, Gq activates phospholipase Cβ, and G12/13 stimulates the guanine nucleotide exchange factor of small GTPases of the Rho family (10). It is predicted that more than 50 GPCRs are expressed in the liver (13). Similar to most other cell types, the functions of HSCs, which have important roles in fibrosis, are also regulated by GPCRs. However, our knowledge of how GPCRs regulate HSCs is insufficient.

This review primarily focuses on the function of GPCRs expressed in HSCs and summarizes the information that could be valuable in uncovering the mechanisms of fibrosis and developing new therapies for NAFLD patients. The receptors discussed in this review were selected by mining the GPCR expression data published by Regard et al. (13) and GPCR-related articles on HSCs in PubMed. To describe the G protein coupling properties of the different GPCRs, we referred to the IUPHAR/BPS Guide to Pharmacology (https://www.guidetopharmacology.org/GRAC/FamilyDisplayForward?familyId=694).

## Role of Gs-Coupled GPCRs in HSCs

### Adrenoceptors

Norepinephrine (NE) and epinephrine (EPI) are released from the sympathetic nerve endings and regulate liver metabolism, among numerous other functions (14). NE and EPI activate Gs-linked hepatic β-adrenoceptors (14). Sigala et al. reported that all three β-adrenoceptor subtypes (β1-3) are expressed in activated human primary HSCs (hHSCs). Furthermore, the expression of β-adrenoceptors in HSCs was increased in the livers of patients with NAFLD cirrhosis (15). At the molecular level, exogenous NE/EPI induced hHSC proliferation in a dose-dependent manner *via* p38 MAP, PI3K, and MEK signaling. NE and EPI increased collagen-1α2 expression *via* transforming growth factor β (TGF-β). These results suggest that hHSCs utilize catecholamines for their survival and fibrotic functions through activation of β-adrenoceptors (15). Similarly, using cultured HSCs and liver-damaged mice, Oben et al. demonstrated that HSCs express α- and β-adrenoceptors and catecholamine biosynthetic enzymes in response to sympathetic stimulation, release NE, and promote liver fibrogenesis (16).

### Dopamine D1 Receptor (DRD1)

Yes-associated protein (YAP) and transcriptional coactivator with PDZ-binding motif (TAZ) have been identified as important factors promoting the activation of mesenchymal cells in human fibrosis (17). Recently, the Gs-coupled dopamine D1 receptor (DRD1) was found to be preferentially expressed in mesenchymal cells of the lung and HSCs in the liver (18). DRD1 stimulation selectively inhibited cellular YAP/TAZ function, shifted the cell phenotype from profibrotic to fibrosis resolving, and ameliorated liver fibrosis in mice (18). Although further studies in the human liver are needed, targeting YAP/TAZ *via* DRD1 could prove a useful pharmacological and cell-selective approach to reverse liver fibrosis.

### Adenosine A2A Receptor (A2-AR)

The adenosine A2A receptor (A2-AR) is a Gs -coupled receptor expressed on rat and human HSCs (19). Adenosine is released from injured tissues, and upon stimulation by adenosine, the A2A-AR promotes collagen production by HSCs (19). Chan et al. found that A2-AR-deficient mice are protected from the development of liver fibrosis after exposure to CCl4 or thioacetamide (19). The use of the adenosine receptor antagonists such as caffeine or ZM241385 also reduced liver fibrosis in wild-type mice exposed to CCl4 or thioacetamide in the same study. These data indicate that hepatic A2-AR plays an active role in the pathogenesis of liver fibrosis (19). The same group reported that A2-AR stimulation promotes collagen expression by HSCs through pathways linking protein kinase A, src, and ERK 1/2 or p38 MAP kinase signaling pathways (20). In addition, adenosine acts as a physiological inhibitor of the Rho pathway and has also been proposed to promote the contraction of HSCs (21). On the basis of these studies, A2-ARs represent a potential target for drug discovery in liver fibrosis.

### Parathyroid Hormone 1 Receptor (PTH1R)

Parathyroid hormone-like hormone (PTHLH) is a cytokine-like polyprotein that is involved in the activation of HSCs in conjunction with TGF-β (22). PTHLH activates HSCs overexpressing TGF-β, and TGF-β promotes the differentiation of HSCs into collagen-producing myofibroblasts (22). When PTHLH was overexpressed in the liver in mice by gene delivery with an adeno-associated virus, spontaneous development of liver fibrosis was observed (23). At the molecular level, PTHLH increased the activation of the hedgehog (Hh) pathway through the Gs-coupled receptor PTHLH 1receptor (PTH1R), causing the activation of HSCs (23).

### Relaxin Family Peptide Receptors 1 (RXFP1) and 2 (RXFP2)

The mammalian hormone relaxin (RLN) is a potential inhibitor of liver fibrosis by stimulating a GPCR known as relaxin family peptide receptor 1 (RXFP1) (24, 25). Specifically, RLN down-regulates collagen-I and TIMP-1, while upregulating interstitial collagenase (MMP-1 in humans) (25). This study also found that the expression of RXFP1 is up-regulated in myofibroblasts/activated HSCs in human fibrotic liver and rat fibrotic liver injury models (25). Another study showed that the Gs/Gi-coupled relaxin family peptide receptor 2 (RXFP2) is also highly expressed in cirrhotic liver (26). Similar to RXFP1, RXFP2 is likely to be involved in the activation of HSCs but the mechanism by which this occurs has not been investigated (26).

### Prostaglandin E Receptor 2 (EP2)

The prostaglandin E2 (EP2) receptor is a Gs-coupled receptor that is activated by prostaglandin E2 (PGE2) (27). Experiments using immortalized human HSCs (LX-1) and primary HSCs suggest that cyclooxygenase-2 (COX-2)-derived PGE2 inhibits both the basal and TGF-β-mediated induction of collagen synthesis (27). However, the role of COX-2-dependent prostaglandins in liver fibrosis is controversial. As mentioned above, there is some evidence that PGE2 inhibits the development of hepatic fibrosis, while other studies have shown that COX-2-dependent prostaglandins promote the development of NASH and cirrhosis (27–31). These discrepant results might be due to the different experimental models used (32). Moreover, the relevance of hepatic EP2 receptors in humans has not been clarified yet.

### Sphingosine-1-Phosphate Receptor 2 (S1PR2)

Sphingosine-1-phosphate receptor 2 (S1PR2)-mediated signaling includes Gs-, Gq-, and G12/13-dependent mechanisms (33). It has been suggested that sinusoidal vasoconstriction, in which HSCs act as a contractile apparatus, plays an important role in the pathophysiology of portal hypertension (33). Previous reports suggested that sphingosine 1-phosphate (S1P) stimulates HSC contractility and increases portal pressure by activating Rho *via* S1PR2 (33). A recent study reported that melatonin inhibits HSC activation *via* the sphingosine kinase 1/S1P system (34). The authors reported that both sphingosine-1-phosphate receptor 1 (S1PR1) and sphingosine-1-phosphate receptor 3 (S1PR3) were associated with liver fibrosis (34). S1PR2 may be involved in bile acid-mediated lipid metabolism in hepatocytes although the molecular mechanisms through which S1PR2 affects hepatocyte and HSC function remains to be investigated (8).

## Role of Gi-Coupled GPCRs in HSCs

### Cannabinoid Receptors 1 (CB1) and 2 (CB2)

Cannabinoids are the active components of marijuana and act through two Gi-coupled GPCRs, cannabinoid receptor 1 (CB1) and cannabinoid receptor 2 (CB2) (35). CB1 is the most abundant receptor in the mammalian brain but is also expressed in peripheral tissues, including various cell types of the liver (36). In a mouse model of liver failure, activation of CB1 on HSCs caused liver failure, and blocking CB1 slowed this process (35). The therapeutic efficacy of CB1 blockers is limited by neuropsychiatric side effects, but the use of novel CB1 antagonists limited to the periphery may overcome such limitations.

CB2 is expressed predominantly by immune and hematopoietic cells (37). Julien et al. demonstrated that CB2 is not detected in normal liver, but it is significantly expressed in non-parenchymal cells in liver biopsy specimens from cirrhotic patients (37). These authors also showed that CB2 is strongly expressed by cultured hepatic myoblasts and activated HSCs. At the molecular level, activation of CB2 caused growth inhibition and apoptosis of these cells, suggesting that CB2 exhibits an anti-fibrotic effect (37). In addition, mice lacking CB2 showed enhanced liver fibrosis when chronically treated with CCl4, when compared to wild-type mice (37). These data suggest that the anti-fibrotic function of CB2 in chronic liver injury could by exploited for therapeutic purposes.

### C-C Chemokine Receptor (CCR)

The inflammatory response to hepatocyte injury plays an important role in the activation of HSCs and liver fibrogenesis (38). When hepatocyte injury occurs, bone marrow-derived monocytes and macrophages are mobilized to the injury site, and activation of resident macrophages (*i.e.*, Kupffer cells) occurs (38). The infiltrating monocytes/macrophages then amplify this immune response by producing pro-inflammatory cytokines and chemokines, which further promote the mobilization of inflammatory cells and upregulate the activation of HSCs (38, 39). Fibrogenic cytokines (such as TGF-β) produced by activated macrophages promote the differentiation of HSCs into myofibroblasts, which form scar-forming matrix proteins such as fibrillar collagen types 1 and 3 and the contractile protein α-SMA, leading to progressive liver fibrosis (38, 40). HSCs express the Gi-coupled receptors C-C chemokine receptor 2 (CCR2) and C-C chemokine receptor 5 (CCR5) (40). There is growing evidence that CCR2/CCR5 and its ligands, including MCP-1 (CCL2) and RANTES (CCL5), are involved in the pathogenesis of liver fibrosis through the promotion of monocyte/macrophage mobilization and tissue infiltration, and the activation of HSCs after liver injury (41–44).

To understand the function of CCR2 in HSCs, Seki et al. performed bile duct ligation (BDL) in mice and showed that CCR2 are strongly expressed in Kupffer cells and HSCs but not in hepatocytes (44). In the same study, BDL- and CCl4-induced liver fibrosis, as assessed by collagen deposition, αSMA expression, and hydroxyproline content of the liver, was markedly reduced in CCR2-deficient mice. Using CCR2 chimeric mice, these authors also found that the fibrotic response required CCR2 expression in resident hepatocytes, including HSCs, but not in Kupffer cells (44). *In vitro* experiments showed that HSCs lacking CCR2 or its downstream mediator p47phox do not exhibit phosphorylation of ERKs or protein kinase B (AKT), chemotaxis, or generation of ROS in response to CC chemokines such as MCP-1 (CCL2), MCP-2 (CCL8), or MCP-3 (CCL7) (44). These results indicate that CCR2 promotes chemotaxis of HSCs and the development of liver fibrosis.

Another study by Seki et al. addressed the function of C-C chemokine receptor 5 (CCR5) in HSCs (42). CCR5 was strongly expressed in cirrhotic human liver and experimental mouse models of fibrogenesis. Further, hepatic fibrosis was greatly reduced in mice treated with the CC chemokine inhibitor 35k or mice lacking CCR5. At a molecular level, CCR5 promoted HSC migration *via* a PI3K-dependent pathway, and CC chemokine-induced migration was strongly suppressed in HSCs lacking CCR5. These data suggest that CCR5 in HSCs contributes to increased fibrosis, similar to CCR2 (42). On the basis of these findings, CCR2 and CCR5 have become attractive targets for antifibrotic therapy (40, 45). In line with this, cenicriviroc, an oral dual CCR2/CCR5 antagonist, has demonstrated anti-fibrotic effects in a thioacetamide-induced rat liver fibrosis model and in a mouse model of diet-induced NASH (40). Cenicriviroc was in a phase III clinical trial in patients with NASH, which was very recently discontinued (NCT03028740) (46).

HSCs also express C-X-C motif receptor 4 (CXCR4) *in vivo* and *in vitro* (47). CXCR4 is activated by stromal cell-derived factor-1 (SDF-1α), an endogenous ligand of CXCR4 (47). The ERK1/2 and phosphoinositide 3-kinase (PI3K) pathways mediate the effects of SDF-1α on HSC collagen-I expression and proliferation (47).

In contrast to other CXRs, the C-X-C motif receptor 3 (CXCR3) has been shown to inhibit liver fibrosis (48). CXCL9, a ligand for CXCR3, exhibited anti-fibrotic effects and suppressed collagen production in LX-2 cells (48). In CXCR3-deficient mice, liver fibrosis was enhanced, and fibrosis progression was associated with a decrease in the number of intrahepatic interferon-γ-positive T cells and a reduction in interferon-γ mRNA (48). These data clearly indicate that CXCL9-CXCR3 regulates Th1-related immune pathways. In the light of these findings, stimulation of CXCR3 by CXCL9 might prove beneficial as an anti-fibrotic therapy.

### Adenosine A3 Receptor (A3-AR)

The A3 adenosine receptor (A3-AR) is a Gi-coupled receptor and is highly expressed in the liver affected by hepatitis (49). Namodenoson, a selective agonist of A3-AR, induces robust anti-inflammatory effects in the liver *via* deregulation of the Wnt/β-catenin pathway (49). The effects of namodenoson in NASH were also investigated using a mouse model of NASH (STAM model), CCl4 fibrotic mice, and in LX-2 cells (50). In the STAM model, namodenoson significantly reduced the NAFLD activity score (NAS) and showed anti-inflammatory and anti-steatotic effects. In the CCl4 fibrosis mouse model, namodenoson significantly improved the degree of hepatic inflammation and fibrosis. Furthermore, namodenoson regulated the Wnt/β-catenin pathway and decreased PI3K expression in liver extracts in CCl4-treated mice and LX2-cells. Overall, these results indicate that namodenoson exerts a protective effect for NASH through the regulation of the PI3K/NF-κB/Wnt/β-catenin signaling pathway (50). Targeting A3-ARs may represent a new direction in the pharmacotherapy of NASH. In fact, a phase 2 clinical trial of namodenoson is currently underway in Israel for the treatment of patients with NASH (NCT04697810).

### G Protein-Coupled Estrogen Receptor 1 (GPER)

The biological effects of estrogen are mediated by two intracellular/nuclear estrogen receptors (ERs; Erα and ERβ) and a transmembrane receptor (G protein-coupled estrogen receptor 1; GPER) (51). These ER subtypes act on cells in different ways and exert different biological responses. Previous studies indicated that estrogen therapy can ameliorate liver fibrosis and inhibit HSC activation through nuclear, ER-dependent changes. Yet, the relationship between GPER and liver fibrosis is unknown (51). Interestingly, a recent study reported that tamoxifen, a drug widely used in the treatment of breast cancer and an agonist of GPER, promotes mechanical deactivation of HSCs *via* the GPER/RhoA/myosin axis (52). This GPER-dependent HSC inactivation system provides new insight into the anti-fibrotic effects of tamoxifen.

### G-Protein Coupled Receptor 91 (GPR91)

Succinate is an essential intermediate of the tricarboxylic acid cycle (53). Succinate binds to G-protein coupled receptor 91 (GPR91, succinate receptor 1) and activates HSCs, induces HSC proliferation and migration, and attenuates HSC apoptosis (53). Succinate-treated mice showed significant molecular changes, including increased production of α-SMA, type 1 collagen, and inflammatory cytokines such as IL-6 and TNF-α (53). Inhibiting the accumulation of succinate might be an effective way to reverse liver fibrosis by inhibiting HSC survival and proliferation. Indeed, it has been shown that metformin inhibits HSC activation by activating the AMPK pathway and inhibiting the succinate-GPR91 pathway (54).

### Somatostatin Receptor (SSTR)

Somatostatin exerts its effects by binding to a family of five Gi-coupled somatostatin receptors (SSTR1 to SSTR5) (55). HSCs of cirrhotic livers and culture-activated HSCs express all five SSTRs, whereas SSTRs are not detected in HSCs of the normal liver (55, 56). Interestingly, an SSTR1 agonist, L-797,591, decreases the migration of HSCs but does not affect HSC proliferation or apoptosis (55). Another study suggests that the effect of octreotide, a SSTR2/5 agonist, on liver fibrosis depends on the cytokine microenvironment of HSCs (57). Thus, our knowledge of SSTR regulation of HSC function is still limited and further studies are needed.

### Neuropeptide Y Receptor Y1 (Y1-R)

Neuropeptide Y (NPY) is a neuropeptide that is abundant in the central and peripheral nervous systems of mammals (58). In cirrhotic patients, serum levels of NPY are elevated and positively correlated with the Model for End-Stage Liver Disease (MELD) score (59). Moreover, the expression levels of NPY and the corresponding Gi-coupled NPY receptor Y1 (Y1-R) were enhanced in activated LX-2 cells (15, 59). At the molecular level, both endogenous and exogenous NPY induced phosphorylation of mTOR, p70S6K, and 4EBP1, thus promoting fibrotic responses in HSCs *via* Y1-R activation. These responses were inhibited by a Y1-R antagonist (BIBP3226) or Y1-R knockdown (59). These results are indicative of an NPY-Y1-R-mediated fibrotic mechanism in HSCs.

### Lysophosphatidic Acid Receptor 1 (LPAR1)

The *Lpar1* gene encodes lysophosphatidic acid receptor 1 (LPAR1), a GPCR that binds to the lipid signaling molecule lysophosphatidic acid (LPA) (60). Previous studies have shown that LPAR1 is expressed in activated HSCs, but minimal expression has been reported in hepatocytes (61). Using single-cell RNA sequencing of healthy and fibrotic mice, Dobbie et al. demonstrated that HSCs consist of topologically distinct lobular regions called portal vein-associated HSCs (PaHSCs) and central vein-associated HSCs (CaHSCs) (60). These authors identified the LPAR1 as a potential therapeutic target for collagen-producing CaHSCs. LPAR1 blockade inhibits liver fibrosis in a murine NASH model which is a finding of potential clinical relevance (60).

### Smoothened Receptor (SMO)

The hedgehog (Hh) signaling pathway regulates the hepatic progenitor cells and liver development (62). Hh signaling includes the Gi- or G12/13-coupled receptor, smoothened (SMO) (63). Although Hh activation has been observed in patients with NAFLD, evidence related to a role of SMO in HSC function is sparse (63). A recent study showed that the Hh pathway regulates HSC-mediated angiogenesis in the liver demonstrating that liver angiogenesis and fibrogenesis are accompanied by SMO and upregulation of hypoxia-inducible factor-1α (HIF-1α) (64). Interestingly, heat shock protein 90 (HSP90) was characterized as a direct target gene for Hh signaling in HSCs (64). Selective inhibition of Hh signaling in HSCs may inhibit fibrosis progression in NAFLD/NASH. Clearly, this pathway is in need of more detailed investigation.

### Frizzled Receptor (Fz)

Wnt signaling is essential for development and implicated in tumorigenesis (65). Wnt ligands bind to the Gi-coupled frizzled receptor (Fz) to transmit downstream signals (65). The expression of Fz2 Wnt4 and Wnt5 ligands were upregulated in activated rat HSCs compared with quiescent rat HSCs in a DNA microarray study (65). Similar findings were obtained in fibrotic livers in mice (65). Further, the increased expression of Wnt5a and its receptor Fz2 indicated that the Wnt/Fz pathway is involved in the differentiation of quiescent HSCs into myoblasts (65). Similar results have been reported by other investigators (66). Wnt signaling could therefore play an important role in the development of liver fibrosis.

### C5a Receptor (C5aR)

C5a is an important component of complement system, a potent chemokine that regulates cell migration in the innate immune system (67). The receptor for C5a, the Gi-coupled C5a receptor (C5aR), is an important regulator of liver immunity and fibrosis (67). Although C5aR expression was detected in fibrotic mice, C5a did not directly affect HSC activation itself but, interestingly, affected HSC migration (68). These data suggest a new mechanism by which the complement system contributes to liver fibrosis.

### Apelin Receptor (APJ)

Immunohistochemical analysis of human liver samples showed that Apelin receptor (APJ) is almost absent in normal livers, while HSCs in cirrhotic livers showed a high expression of APJ (69, 70). *In vitro*, sustained hypoxia and lipopolysaccharide promoted APJ expression in LX-2 cells (70). In turn, activation of APJ promoted the expression of angiopoietin-1 and cell survival in LX-2 cells (70). These results suggest that hypoxia and inflammatory factors play a major role in the activation of the apelin system in HSCs, which triggers angiogenic and proliferative responses in chronic liver disease.

### M2 and M3 Muscarinic Acetylcholine Receptors

The neurotransmitter acetylcholine (ACh) plays a role in hepatic fibrogenesis (71). Expression of the M2 muscarinic ACh receptor (M2) is enhanced in human NASH livers as fibrosis progresses (71). Exogenously administered Ach induces hHSC hyperproliferation and is accompanied with upregulation of fibrotic markers, such as TGF-β and COL1A2 gene expression (71). Ach exerts these effects in HSCs *via* M2 (Gi-coupled) and M3 (Gq-coupled) acetylcholine receptors by activating PI3K and MEK pathways (71). Further, cell proliferation and expression of fibrotic markers are inhibited upon pharmacological inhibition of M2 and M3 receptors, suggesting that suppression of these receptors may lead to inhibition of fibrosis (71).

## Role of Gq-Coupled GPCRs in HSCs

### Angiotensin II Type I Receptor (AT1R)

The role of Gq coupled angiotensin II type I receptor (AT1R) is relatively well investigated in the liver. In patients with chronic liver diseases including NASH, the renin-angiotensin system (RAS) is reported to be activated (72). In line with this finding, angiotensin II induces HSC proliferation and increases TGF-β expression *via* AT1R (72, 73). Furthermore, angiotensin II was reported to induce hepatic fibrosis through the Janus kinase 2 (JAK2)-mediated intracellular action in HSCs (74). These authors also showed that stimulation of AT1R in wild-type mice resulted in JAK2 phosphorylation and activation of RhoA, and Rho-associated kinase 1 (ROCK1), leading to HSC activation and fibrosis (74). By contrast, these effects were blocked in AT1R-deficient mice, indicating that AT1R signaling may promote fibrosis (74).

Meta-analysis of the effects of angiotensin-converting enzyme inhibitors and angiotensin receptor blockers on patients with liver fibrosis showed a reduction in serum hepatic fibrosis markers such as TGF-β, TIMP-1, MMPs, and collagen (75). Additionally, in a randomized, open-label, controlled trial in compensated patients with alcoholic liver fibrosis (F2 or higher), an AT1R blocker, candesartan, significantly reduced histological fibrosis scores and decreased expression of αSMA, TGF-β, TIMP-1, and MMPs (76). Thus, both animal and human studies have shown the efficacy of angiotensin-AT1R blocking drugs in improving fibrosis; however additional clinical trials are needed to ensure the safe and efficient use of these agents in the treatment of liver fibrosis.

### Serotonin Receptor (5-HT)

Rat and human HSCs express several Gq-coupled serotonin receptor subtypes, including serotonin receptor 1B (5-HT1B), serotonin receptor 2A (5-HT2A), and serotonin receptor 2B (5-HT2B). Interestingly, the expression of these receptors is upregulated during HSC activation (77). Antagonizing 5-HT2A in thioacetamide-treated rats and immortalized human HSCs inhibits fibrosis and induces apoptosis (78). Stimulation of 5-HT2B in HSCs has been shown to upregulate the expression of TGF-β, a potent inhibitor of hepatocyte proliferation, and inhibit hepatocyte regeneration (79). Hepatocyte proliferation is enhanced in liver injury models that selectively antagonize 5-HT2B, in mice lacking 5-HT2B, and in wild-type mice that are selectively depleted of HSCs (79). Additionally, 5-HT2B antagonism reduces liver fibrosis in mice and improves liver function (79).

### Arginine Vasopressin Receptor 1A (AVPR1A)

The arginine vasopressin receptor 1A (AVPR1A), which mediates the potent vascular contractile actions of arginine vasopressin (AVP), is also expressed in HSCs (80). AVP increases intracellular calcium concentrations and induces contraction in HSCs in a dose-dependent manner (80). In addition, AVP increases MAPK activity, DNA synthesis, and the number of HSCs in the same experimental settings (80). These effects are similar to those observed in vascular smooth muscle cells and are inhibited by AVPR1A antagonists (80).

### Endothelin Receptor (ETR)

Endothelin (ET) has been implicated in the regulation of hepatic microcirculation and the development of portal hypertension (81). Expression of the Gq-coupled ET receptor type A (ETAR) was up-regulated in HSCs by endotoxin *via* both vasorelaxant nitric oxide (NO)-dependent and NO-independent pathways (82). An endotoxin-ETAR interaction could therefore be important in acute endotoxemia and chronic liver injury.

On the other hand, immunohistochemical studies in normal human liver tissues showed that ET receptor type B (ETBR; coupling profile: Gs/Gi/Gq) is predominantly expressed in HSCs, while ETAR is poorly expressed in these cells (81). The expression of ETBR was significantly increased in HSCs of cirrhotic livers, while ETAR expression was increased to a considerably lower degree (81). These data suggest that the increased expression of ETBR in the cirrhotic liver may enhance the effect of endothelin on HSCs and increase hepatic microvascular tone.

### G Protein-Coupled Receptor 55 (GPR55)

GPR55 is considered to be a putative Gq- or G12/13-coupled receptor for cannabinoids in addition to the classical CB1 and CB2 receptors. l-α-Lysophosphatidylinositol (LPI) is the only known endogenous ligand for GPR55 (83). GPR55 has been implicated in energy homeostasis in various organs (83). A recent study showed that LPI blood levels and GPR55 expression in the liver were elevated in NASH patients (84). Further, LPI increased lipid content in human hepatocytes and mouse liver by inducing activation of the acetyl-coenzyme A carboxylase (ACC) *via* adenosine monophosphate-activated protein kinase, thus inducing *de novo* lipid synthesis and decreasing beta-oxidation (84). Inhibition of GPR55 and ACCα inhibited the action of LPI, and knockdown of GPR55 *in vivo* was sufficient to ameliorate liver damage in mice fed a high-fat diet or a methionine-choline-deficient diet (84). Furthermore, LPI promoted the initiation of HSC activation by stimulating GPR55 and activating ACC. These findings suggest that the LPI/GPR55 system is involved in the pathogenesis of NAFLD/NASH by activating ACC (84).

### Protease-Activated Receptor-2 (PAR2)

Protease-activated receptor-2 (PAR2) is activated by serine proteases and activated coagulation factors (85). PAR2 couples to multiple G proteins (Gq, Gi, and G12/13) (85). In mice, deletion of PAR2 suppressed the progression of CCl4-induced liver fibrosis (86). PAR2 has been shown to stimulate activation and proliferation, collagen production, and TGF-β protein production in human HSCs (86).

## Role of G12/13 Signaling in HSCs

Experiments with HSCs have shown that G12 and G13 regulate TGF-β gene expression *via* a Rho/Rac-dependent increase in activating protein 1 activity (87). Interestingly, G12 is overexpressed in activated HSCs and fibrotic liver (88). In a mouse model of liver fibrosis induced by CCl4, deletion of G12 suppressed fibrosis and liver damage (88). This effect was attenuated by a lentivirus that introduced G12 into HSCs. The activation of G12 promoted autophagy with c-Jun N-terminal kinase-dependent ATG12-5 conjugation. Furthermore, miR-16 directly inhibited the *de novo* synthesis of G12 and altered autophagy in HSCs (88). These results suggest that dysregulation of miR-16 in HSCs leads to overexpression of G12 and activates HSCs by promoting autophagy (88).

## Closing Remarks

**Figure 3:**
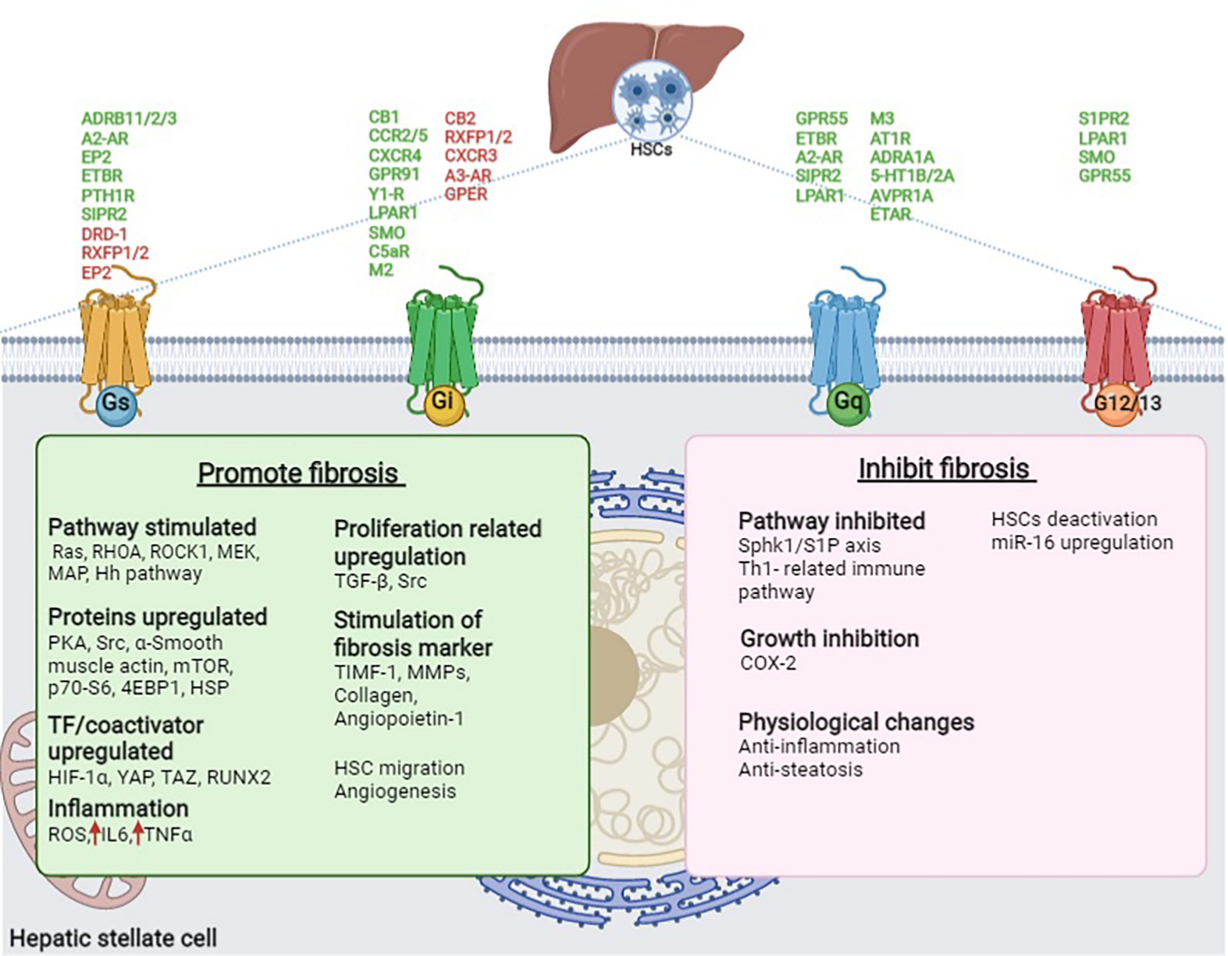
GPCRs present in HSCs involved in promoting and inhibiting fibrosis in the liver. GPCR (Green- promote fibrosis and Red- inhibits fibrosis), Ach, Acetylcholine; ADRB1/2/3, β1/2/3-adrenoceptor; AVPR1A, Arginine vasopressin receptor 1A; AT1R, Angiotensin II type I receptor; A2-AR, Adenosine A2A receptor; CB1/2, Cannabinoid receptor 1/2; CCR2/5, C-C chemokine receptor type 2/5; COX2, Cyclooxygenase 2; CXCR3/4, C-X-C motif chemokine receptor 3/4; C5aR, C5a receptor; C5a, Complement peptide; DRD1, Dopamine receptor D1; EP2, Prostaglandin E receptor 2; EPI, Epinephrine; ETAR/BR, Endothelin receptor type A/B; ETBR, Endothelin receptor type A; Fz2, Frizzled receptor 2; GPR91, G protein-coupled bile acid receptor 91; GPR55, G protein-coupled receptor 55; GPER, G protein-coupled estrogen receptor 1; Hh, Hedgehog; HIF-1α, Hypoxia-inducible factor 1- α; HSC, Hepatic stellate cell; HSP, Heat shock proteins; IL6, Interleukin-6; LA, Lysophosphatidic acid; LPAR1, Lysophosphatidic acid receptor 1; MAPK, mitogen-activated protein kinases; miR-16, microRNA 16; MEK, Mitogen-activated protein kinase; MMP, Matrix metalloproteinases; mTOR, mammalian target of rapamycin; M2/3, M2/3 acetylcholine receptor; NE, Norepinephrine; NPY, Neuropeptide Y; PKA, Protein kinase A; PGE2, Prostaglandin E2; PTHLH, Parathyroid Hormone Like Hormone; PTH1R, Parathyroid hormone 1 receptor; RHOA, Ras homolog family member A; RLN, Relaxin; ROCK, Rho-associated protein kinase; ROS, reactive oxygen species; RUNX2, Runt-related transcription factor 2; RXFP1/2, Relaxin family peptide receptor 1/2; SMO, Smoothened receptor; S1PR2, Sphingosine-1-phosphate receptor 2; TAZ, PDZ-binding motif; TGF-β. Transforming growth factor-beta; TIMF-1, Thymocyte Interaction Modulation Factor; 5-HT1B/2A, Serotonin receptor 1B/2A; p70-S6, S6 kinase beta-1; 4EBP1, 4E binding protein; Sphk1/S1P, sphingosine kinase-1/sphingosine-1-phosphate; YAP, yes-associated protein 1; Y1-R, Neuropeptide Y receptor Y1.

**Table 1** and **Figure 3** summarize the GPCRs present in HSCs that are predicted to be involved in the promotion and suppression of liver fibrosis, as described in this manuscript. It is interesting to note that most of the GPCRs in HSCs are involved in activating liver fibrosis; however, some of the receptors contribute to the suppression of fibrosis. Theoretically, suppression of fibrosis-promoting receptors or stimulation of fibrosis-suppressing receptors in HSCs could lead to the treatment or prevention of liver fibrosis. Clearly, multiple factors are intervening in the modification of GPCR action in HSCs, including the activity of GPCRs expressed in hepatocytes, biliary epithelial cells, vascular endothelial cells, and various immune cells in the liver, as well as the activity of extrahepatic GPCRs. Furthermore, in addition to the receptors discussed above, many other GPCRs are present in HSCs, including many orphan receptors (13). The potential roles of these receptors in regulating hepatic fibrosis remain to be explored. In conclusion, an improved understanding of the functions of GPCRs in HSCs may lead to the development of novel drugs that could prove clinically useful for the treatment of chronic liver disease, NAFLD, and NASH.

**Table 1:** Fibrotic function of G protein-coupled receptors expressed in HSCs.

Receptor name	Family	Effect on liver fibrosis	Primary Transduction Mechanisms			Reference
β1-adrenoceptor (ADRB1)	Adrenoceptors	**⇧**	Gs			(15, 16)
β2-adrenoceptor (ADRB2)	Adrenoceptors	**⇧**	Gs			(15)
β3-adrenoceptor (ADRB3)	Adrenoceptors	**⇧**	Gs			(15)
Dopamine receptor D1 (DRD1)	Dopamine receptors	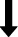	Gs			(18)
Adenosine A2A receptor (A2-AR)	Adenosine receptors	**⇧**	Gs	Gq		(19)
Parathyroid hormone 1 receptor (PTH1R)	Parathyroid hormone receptors	**⇧**	Gs			(23)
Relaxin family peptide receptor 1 (RXFP1)	Relaxin family peptide receptors	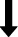	Gs	Gi		(26)
Relaxin family peptide receptor 2 (RXFP2)	Relaxin family peptide receptors	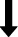	Gs	Gi		(26)
Prostaglandin E receptor 2 (EP2)	Prostanoid receptors	**⇧** 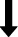	Gs			(27)
Sphingosine-1-phosphate receptor 2 (S1PR2)	Lysophospholipid (S1P) receptors	**⇧**	Gs	Gq	G_12/13_	(33)
Endothelin receptor type A (ETBR)	Endothelin receptors	**⇧**	Gs	Gi	Gq	(81)
Cannabinoid receptor 1 (CB1)	Cannabinoid receptors	**⇧**	Gi			(36)
Cannabinoid receptor 2 (CB2)	Cannabinoid receptors	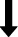	Gi			(36)
C-C chemokine receptor type 2 (CCR2)	Chemokine receptors	**⇧**	Gi			(40)
C-C chemokine receptor type 5 (CCR5)	Chemokine receptors	**⇧**	Gi			(40)
C-X-C motif chemokine receptor 3 (CXCR3)	Chemokine receptors	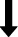	Gi			(48, 49)
C-X-C motif chemokine receptor 4 (CXCR4)	Chemokine receptors	**⇧**	Gi			(48, 49)
Adenosine A3 receptor (A3-AR)	Adenosine receptors	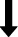	Gi			(49)
G protein-coupled estrogen receptor 1 (GPER)	G protein-coupled estrogen receptor	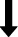	Gi			(51)
G protein-coupled bile acid receptor 91 (GPR91)	Succinate receptors	**⇧**	Gi			(54)
Neuropeptide Y receptor Y1 (Y1-R)	Neuropeptide Y receptors	**⇧**	Gi			(15, 59)
Lysophosphatidic acid receptor 1 (LPAR1)	Lysophospholipid receptors	**⇧**	Gi	Gq	G_12/13_	(60)
Smoothened receptor (SMO)	Class Frizzled GPCRs	**⇧**	Gi	G_12/13_		(63)
Frizzled receptor 2 (Fz2)	Class Frizzled GPCRs	**⇧**	Gi			(65)
C5a receptor (C5aR)	Complement peptide receptors	**⇧**	Gi			(67)
Apelin receptor (APJ)	Apelin receptor	**⇧**	Gi			(70)
M2 acetylcholine receptor (M2)	Acetylcholine receptors (muscarinic)	**⇧**	Gi			(71)
M3 acetylcholine receptor (M3)	Acetylcholine receptors (muscarinic)	**⇧**	Gq			(71)
Angiotensin II type I receptor (AT1R)	Angiotensin receptors	**⇧**	Gq			(72, 73)
α1A-adrenoceptor (ADRA1A)	Adrenoceptors	**⇧**	Gq			(59)
Serotonin receptor 1B (5-HT1B)	5-Hydroxytryptamine receptors	**⇧**	Gq			(77)
Serotonin receptor 2A (5-HT2A)	5-Hydroxytryptamine receptors	**⇧**	Gq			(77)
Arginine vasopressin receptor 1A (AVPR1A)	Vasopressin and oxytocin receptors	**⇧**	Gq			(80)
Endothelin receptor type A (ETAR)	Endothelin receptors	**⇧**	Gq			(82)
G protein-coupled receptor 55 (GPR55)	GPR18, GPR55 and GPR119	**⇧**	Gq	G_12/13_		(83)

## Author Contributions

TK and SS collected the information and prepared the manuscript. NT and TU supervised the design and content of this paper. All authors contributed to the article and approved the submitted version.

## Funding

This research was supported in part by the Intramural Research Program of the NIH, The National Institute of Diabetes and Digestive and Kidney Diseases (NIDDK).

## Conflict of Interest

The authors declare that the research was conducted in the absence of any commercial or financial relationships that could be construed as a potential conflict of interest.

## Publisher’s Note

All claims expressed in this article are solely those of the authors and do not necessarily represent those of their affiliated organizations, or those of the publisher, the editors and the reviewers. Any product that may be evaluated in this article, or claim that may be made by its manufacturer, is not guaranteed or endorsed by the publisher.
